# Inhibitory Effects and Mechanisms of Volatile Organic Compounds from *Schizophyllum commune* Against the Pepper Gummosis Pathogen *Fusarium tricinctum*

**DOI:** 10.3390/antiox15040437

**Published:** 2026-03-31

**Authors:** Bin Wang, Yuke Yan, Yuyan Sun, Chongqing Zhang, Xinyi Wang, Wei Chen, Jing He

**Affiliations:** 1 College of Forestry, Gansu Agricultural University, Lanzhou 730070, China; wangbin@gsau.edu.cn (B.W.); xlsyyk@163.com (Y.Y.); 13034101180@163.com (Y.S.); zhangchongqing2022@163.com (C.Z.); 15374492393@163.com (X.W.); 18298345663@163.com (W.C.); 2 Wolfberry Harmless Cultivation Engineering Research Center of Gansu Province, Lanzhou 730070, China

**Keywords:** *Schizophyllum commune*, *Fusarium tricinctum*, volatile organic compounds, mushroom alcohol, biological control

## Abstract

Background: Gumming disease caused by *Fusarium tricinctum* severely threatens *Zanthoxylum bungeanum* production. This study investigated the antifungal potential of volatile organic compounds (VOCs) produced by an endophytic fungus, *Schizophyllum commune*, isolated from *Z. bungeanum*. Methods: A dual-culture assay evaluated VOCs inhibition against *F. tricinctum*. Compounds were identified using headspace solid-phase microextraction gas chromatography-mass spectrometry, and the antifungal mechanism of this component was explored. Results: VOCs from *S. commune* significantly inhibited mycelial growth and sporulation of the pathogen. Among 53 identified compounds, 1-octen-3-ol (mushroom alcohol) was the most abundant (35.98% relative content) and exhibited strong antifungal activity with an EC_50_ of 0.15 µL/mL against *F. tricinctum*. Mechanistically, 1-octen-3-ol disrupted cell membrane integrity by increasing alkaline phosphatase and β-1,3-glucanase activities, leading to enhanced permeability and content leakage. It also induced oxidative stress by promoting reactive oxygen species accumulation via elevated NADPH oxidase and superoxide dismutase activities, while suppressing antioxidant enzymes. Conclusions: 1-octen-3-ol inhibits *F. tricinctum* through membrane disruption and oxidative stress, offering a promising eco-friendly strategy for controlling gumming disease.

## 1. Introduction

*Zanthoxylum bungeanum* is an important specialty economic tree and a source of condiments in China. Its cultivation plays a key role in promoting economic development in mountainous areas and increasing farmers’ income [1]. However, in recent years, gumming disease has become widespread and increasingly severe in major *Z. bungeanum* production regions, causing significant economic losses for farmers [2]. Typical symptoms of this disease include the secretion of a gum-like substance from the bark of the trunk or branches, leading to tree weakness, dieback of branches, and in severe cases, death of the entire plant, which significantly reduces the yield and quality [3]. Gumming disease is often caused by the complex infection of multiple pathogenic fungi, among which species of the genus *Fusarium* spp. are an important group of pathogens [4]. *Fusarium tricinctum*, one of the confirmed pathogens of *Z. bungeanum* gumming disease, possesses strong infectivity and a wide adaptability range, and can induce typical gumming symptoms [2]. Currently, the control of this disease still relies on chemical agents. However, long-term use has led to increasingly prominent problems such as increased pathogen resistance, pesticide residues, and environmental pressure [5]. Therefore, developing safe, efficient, and environmentally friendly novel control strategies and elucidating their mechanisms of action are of great significance for ensuring the sustainable development of the *Z. bungeanum* industry.

In recent years, volatile organic compounds (VOCs) released by microorganisms have attracted significant attention as a novel class of bioactive substances for plant disease control [6]. Characterized by high permeability, easy degradability, and high diffusion efficiency, VOCs demonstrate better efficacy when used as biofumigants in relatively enclosed environments [7,8]. They encompass a wide variety of types, including alcohols, esters, ketones, terpenes, and others [9]. Studies have shown that VOCs from various fungi and bacteria possess broad-spectrum antimicrobial activity, affecting mycelial growth, spore germination, and cell membrane integrity [10]. For example, 1-Octen-3-ol was identified as a key antifungal component in VOCs produced by *Trichoderma asperellum* and *Aspergillus niger*, effectively controlling pear Valsa canker [11,12]. Similarly, 2-Octen-1-ol from *Irpex lacteus* [13] and 2-nonanol from Bacillus aryabhattai [14] showed strong inhibitory effects against *Botrytis cinerea* and *Penicillium expansum*, respectively. VOCs from *T. koningiopsis* T-51 also suppressed *B. cinerea* and *F. oxysporum* [15]. These findings highlight the potential of specific VOCs as biocontrol agents. Mechanistic studies have shown that VOCs can disrupt fungal cell membrane integrity and interfere with key metabolic pathways [12,13,14,15]. These insights provide a basis for investigating the mechanisms of action of such compounds.

In a preliminary study, we isolated an endophytic fungus strain HJ-18 from healthy *Z. bungeanum* plants, which exhibited strong antagonistic activity against *F. tricinctum*. This strain was morphologically and molecularly identified as *Schizophyllum commune* (GenBank accession number: OP502072) [4]. *S. commune* is a common basidiomycete with a wide global distribution. Studies have shown that it can produce a diverse array of VOCs, which exhibit inhibitory activity against plant pathogenic fungi, bacteria, and nematodes, suggesting its potential for biological control [16,17]. However, current research on VOCs from *S. commune* has primarily focused on preliminary antimicrobial screening, while systematic identification of its active components and elucidation of their mechanisms of action remain lacking. Therefore, this study aims to systematically evaluate the inhibitory effects of VOCs from *S. commune* against *F. tricinctum*, analyze the VOC composition using SPME-GC-MS, and investigate the antifungal mechanism of the key active component. Although 1-octen-3-ol has been previously reported to exhibit antifungal activity against several plant pathogens, its mode of action remains incompletely understood, particularly regarding the dynamics of reactive oxygen species (ROS) accumulation and the coordinated impact on cell wall integrity. Moreover, its efficacy and mechanism against *F. tricinctum*, the causal agent of pepper gummosis, have not been systematically investigated. In the present study, we therefore aim to provide a comprehensive mechanistic insight into how 1-octen-3-ol affects *F. tricinctum*, focusing on the interplay between cell wall disruption, membrane damage, and oxidative stress. The research findings will provide a theoretical basis and reference for the development of novel biofumigants or biocontrol agents based on *S. commune* or its active VOCs.

## 2. Materials and Methods

### 2.1. Strains and Reagents

*S. commune* HJ-18 was isolated from healthy *Z. bungeanum* plants, while *F. tricinctum* was isolated from *Z. bungeanum* plants affected by gumming disease. Both strains were identified through molecular biology and pathogenicity re-inoculation tests, and are preserved in the Forest Protection Laboratory of the College of Forestry, Gansu Agricultural University. 1-octen-3-ol (mushroom alcohol, CAS: 3391-86-4, purity ≥ 98%) was purchased from Shanghai Yuanye Bio-Technology Co., Ltd. (Shanghai, China, product code: S46133).

### 2.2. Antifungal Assay of S. commune VOCs

The inhibitory effect of *S. commune* VOCs was evaluated through a double Petri dish sealing assay [18]. *S. commune* and *F. tricinctum* were pre-cultured on PDA plates for 7 d. Mycelial plugs (6 mm in diameter) were then taken from the colony edges and inoculated at the center of separate PDA plates (90 mm in diameter). After removing the lids, the two plates were placed face-to-face and sealed together with parafilm. A sterile, breathable glass paper (10 cm × 10 cm) was placed between the two plates to prevent direct contact and cross-contamination while allowing gas exchange. The distance between the two mycelial plugs was approximately 10 mm, determined by the combined height of the two Petri dish bases. The headspace volume of the sealed system was approximately 130 mL (calculated based on the internal dimensions of the 90 mm Petri dishes). To minimize gas leakage, the junction between the two plates was tightly sealed with parafilm, and the breathable glass paper ensured uniform diffusion of VOCs within the confined space. Controls were prepared by sealing a blank PDA plate with a plate inoculated only with F. tricinctum. All setups were incubated in the dark at 25 °C.

At 3, 5, 7, and 9 d of incubation, the colony diameters of *F. tricinctum* in both treated and control groups were measured using the cross method. Mycelia were scraped and weighed for biomass determination. The experiment was repeated three times.

Sporulation was measured according to a previous report [19]. At 3, 5, 7, and 9 d of incubation, 10 mL of sterile water was added to each plate, and spores were gently scraped from the PDA surface using a sterile spreader. The suspension was filtered through four layers of sterile gauze, and the filtrate was collected into a 10 mL centrifuge tube. Conidial numbers were counted using a hemocytometer. The experiment was repeated three times.

### 2.3. VOC Composition Analysis

The compositional analysis of *S. commune* VOCs was performed according to a previous report, using headspace solid-phase microextraction coupled with gas chromatography-mass spectrometry (SPME-GC-MS) [13]. An SPME fiber assembly (250/30 μm divinylbenzene/carboxen) was conditioned at 220 °C for 40 min. Subsequently, a hole (0.5 mm in diameter) was drilled in the side wall of the Petri dish. The SPME fiber was inserted into the dish to adsorb volatiles from the headspace above the culture medium onto the polydimethylsiloxane coating, with an adsorption time of 40 min. This procedure was repeated at least three times for each sample. The fiber containing the adsorbed VOCs was introduced into the injector port of a TRACE DSQ system at 240 °C in splitless mode for thermal desorption. The desorbed compounds were separated on an HP-5MS capillary column. The analytical parameters were as follows: (1) Column: HP-5MS capillary column (30 m × 0.25 mm × 0.25 μm); (2) Oven temperature program: initial temperature held at 50 °C for 2 min, increased to 180 °C at a rate of 5 °C/min and held for 5 min, then increased to 250 °C at 10 °C/min and held for 5 min; (3) Injector temperature: 250 °C; transfer line temperature: 280 °C; carrier gas (He) flow rate: 1 mL/min; (4) Split ratio: splitless; (5) Mass spectrometry conditions: ion source temperature: 230 °C; quadrupole temperature: 150 °C; ionization: electron impact (EI) source at 70 eV; scan rate: 5 spectra/s; mass scan range: 40–600 amu.

The VOCs were tentatively identified by comparing the acquired mass spectra with the NIST 11 database (Scientific Instrument Services, Inc., Ringoes, NJ, USA). Components with a mass spectral match factor > 70% were selected. The relative amount of each component was determined based on peak area percentages, averaged from three replicates. Authentic standards were used for the confirmation of volatile compounds. Volatile components originating from the blank PDA medium itself were excluded from the final analysis. Analysis of variance (ANOVA) followed by Tukey’s test was performed to assess significant differences among the results. The relative amount of each component was determined based on peak area percentages averaged from three replicate injections. Absolute concentrations were not calculated, as no calibration curves were established for individual compounds. Therefore, the reported values reflect the relative abundance of each compound within the VOC profile under the standardized experimental conditions.

### 2.4. Half-Maximal Effective Concentration (EC_50_) Determination of 1-Octen-3-ol

The EC_50_ concentration of 1-octen-3-ol was determined according to a previous report [20]. Based on GC-MS analysis, the commercially available compound 1-octen-3-ol was purchased. Five concentration gradients—0.05, 0.10, 0.15, 0.20, and 0.25 µL/mL—were established based on preliminary concentration screening. The filter paper disk method was used to create corresponding headspace concentrations in PDA plates. A 2.5 µL aliquot of *F. tricinctum* spore suspension (1 × 10^7^ spores/mL) was inoculated at the center of each PDA plate. The plates were sealed and incubated in the dark at 25 °C. After 7 d, colony diameters were measured, and the mycelial growth inhibition rate was calculated as: Inhibition rate (%) = [(D_control − D_treatment)/D_control] × 100, where D_control and D_treatment represent the colony diameters of the control and treatment groups, respectively. The EC_50_ value was calculated by linear regression analysis using the concentration as the independent variable (x) and the inhibition rate as the dependent variable (y).

### 2.5. Antifungal Assay of 1-Octen-3-ol and Mycelium Collection

PDA plates supplemented with the EC_50_ concentration of 1-octen-3-ol were used for the treated group, while plain PDA plates served as the control. *F. tricinctum* was cultured on both types of plates. At 3, 5, 7, and 9 d of incubation, colony diameters were measured using the cross method. Thereafter, mycelium were collected, scraped, and weighed to determine biomass. The collected mycelium were rapidly frozen in liquid nitrogen and then stored in an ultra-low temperature freezer at −80 °C. For each assay, 0.1 g of mycelium was accurately weighed.

### 2.6. Determination of Alkaline Phosphatase (AKP) and β-1,3-Glucanase Activities

The activity of AKP was determined according to previous report and the instructions of the assay kit (Shanghai Youxuan Biotechnology Co., Ltd., Shanghai, China, YX-W-B002) [21]. One unit of enzyme activity was defined as the amount of enzyme that catalyzes the production of 1 μmol of phenol per minute per milligram of protein at 37 °C. AKP activity was expressed as U/g.

The activity of *β*-1,3-glucanase was determined as follows: 0.1 g of *F. tricinctum* mycelium was accurately weighed and homogenized in 0.9 mL of sodium acetate buffer (0.05 M, pH 5.0) in an ice bath. The reaction mixture consisted of 100 μL of the supernatant and 100 μL of laminarin solution (1 mg/mL). After incubation at 37 °C for 30 min, 1 mL of 3, 5-dinitrosalicylic acid solution was added to terminate the reaction. The mixture was then boiled for 5 min to develop color, followed by cooling to room temperature under running water. A control was prepared using enzyme solution inactivated by boiling for 10 min. One unit of enzyme activity was defined as the amount of enzyme required to cause a change in absorbance of 0.01 at 540 nm per hour under the assay conditions. β-1, 3-glucanase activity was expressed as U/mg.

### 2.7. Determination of Conductivity, Protein and Nucleic Acid Leakage

The measurements of conductivity and the leakage of protein and nucleic acids were conducted according to previous report [21]. To determine conductivity, 0.1 g of fresh mycelium was added to deionized water, and the conductivity was measured using a portable conductivity meter after 4 h of incubation. The conductivity were expressed in μS/cm. For the determination of protein and nucleic acid leakage, 0.1 g of mycelium was added to 10 mL of sterile water. After centrifugation, the supernatant was collected, and its absorbance was measured at 280 nm and 260 nm. The OD_280_ and OD_260_ values were used to indicate the relative content of extracellular protein and extracellular nucleic acid materials, respectively.

### 2.8. Determination of Redox Balance-Related Indicators

The generation rate of O_2_^−^ was measured using a Superoxide Anion Content Assay Kit (Solarbio, Beijing, China, BC1290) and expressed as μmol/min/g. The content of H_2_O_2_ was determined using an assay kit (Solarbio, Beijing, China, BC3595) and expressed as μmol/g. The content of hydroxyl radical (·OH) was measured using a kit (Jiancheng Bioengineering Institute, Nanjing, China, A018-1-1). One unit of hydroxyl radical generation capacity was defined as the amount causing a 1 mmol/L increase in the concentration of the reaction mixture per mL of sample, and the result was expressed as U/mL.

NADPH oxidase (NOX) activity was measured using a kit (Jiancheng Bioengineering Institute, Nanjing, China, A116-1-1). One unit of enzyme activity was defined as the amount causing a change of 0.01 in A_600_ per minute per gram of tissue in 1 mL of the reaction system. NOX activity was expressed as U/g. Superoxide dismutase (SOD) activity was determined using a kit (Solarbio, Beijing, China, BC5165). One unit of SOD activity was defined as the amount of enzyme causing 50% inhibition in the coupled xanthine oxidase reaction system, and expressed as U/g. Catalase (CAT) activity was measured using a kit (Solarbio, Beijing, China, BC0200). One unit of enzyme activity was defined as the amount catalyzing the decomposition of 1 μmol of H_2_O_2_ per minute per gram of tissue in the reaction system. CAT activity was expressed as U/g. Peroxidase (POD) activity was determined using a kit (Solarbio, Beijing, China, BC0090). One unit of enzyme activity was defined as the amount causing a change of 0.01 in A_470_ per minute per gram of tissue in 1 mL of the reaction system. POD activity was expressed as U/g. Ascorbate peroxidase (APX) activity was measured using a kit (Solarbio, Beijing, China, BC0220). One unit of enzyme activity was defined as the amount oxidizing 1 μmol of ascorbate (ASA) per minute per gram of tissue. APX activity was expressed as U/g. Glutathione (GSH) content was determined using a kit (Jiancheng Bioengineering Institute, Nanjing, China, A006-1-1) and expressed as mg/g prot. Protein content was measured using the Coomassie Brilliant Blue method with a kit (Jiancheng Bioengineering Institute, Nanjing, China, A045-2) and expressed as g/L.

### 2.9. Statistical Analysis

All experiments were performed with three independent biological replicates. For each biological replicate, measurements were conducted in triplicate, and the mean values were used for subsequent statistical analysis. Statistical analyses were performed using SPSS 24.0. For comparisons between two groups, independent samples *t*-test was employed. For comparisons among multiple groups, one-way analysis of variance (ANOVA) followed by Duncan’s multiple range test was conducted. Graphs were generated using Origin 2021.

## 3. Results

### 3.1. Antifungal Activity of Volatile Compounds from S. commune HJ-18

**Figure 1:**
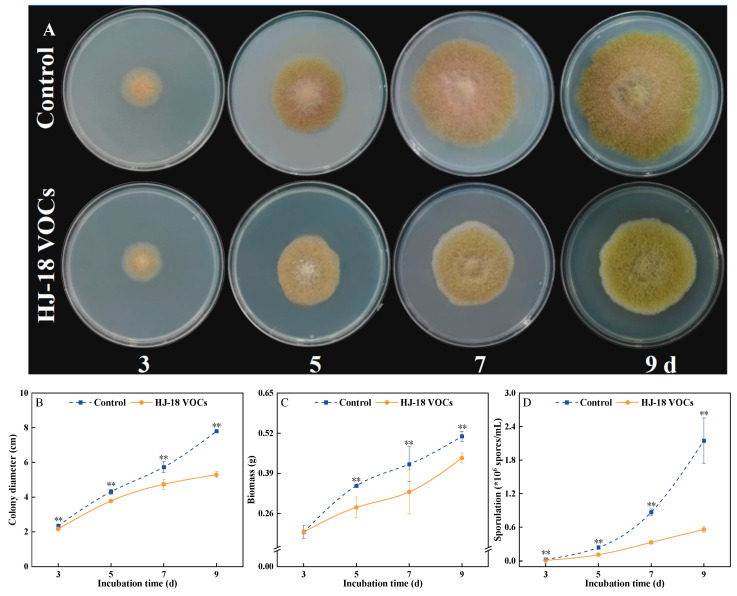
Effect of *Schizophyllum commune* HJ-18 VOCs on colony growth (**A**), diameter (**B**), biomass (**C**), and sporulation (**D**) of *Fusarium tricinctum*. Values are presented as mean ± SE (*n* = 3). ** indicate significant differences at *p* < 0.01.

Colony growth could intuitively reflect the growth status of fungi. The results of the sealed-plate assay showed that the VOCs produced by *S. commune* exhibited a significant inhibitory effect on *F. tricinctum*, which was time-dependent (Figure 1A). In the early stages of incubation, the difference in colony size between the treatment and control groups was minor. As incubation time increased, the inhibitory effect became progressively more pronounced. After 9 d of treatment, the colony diameter of the treatment group was significantly reduced by 32.2% compared to the control group (Figure 1B). Beyond colony morphology, biomass and sporulation data further confirmed this inhibitory effect. Biomass determination results indicated that after 7 d of treatment, the biomass of the treatment group was significantly reduced by 44.12% compared to the control (Figure 1C). Concurrently, VOC treatment exerted a stronger inhibitory effect on the reproductive capacity of *F. tricinctum*, with sporulation significantly reduced by 62.07% compared to the control group after 7 d of treatment (Figure 1D). In summary, the VOCs not only effectively inhibited the growth and biomass accumulation of *F. tricinctum* but also strongly interfered with its sporulation process.

### 3.2. Identification of VOCs from S. commune HJ-18

**Figure 2:**
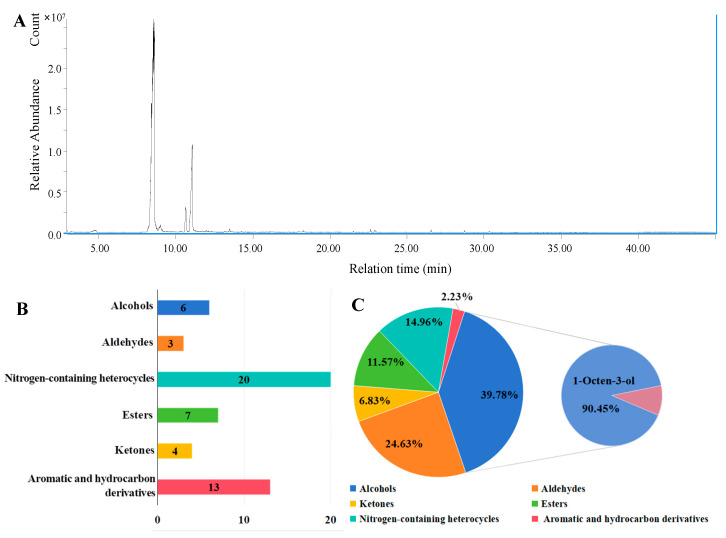
Identification and analysis of VOCs produced by *Schizophyllum commune* HJ-18. (**A**) Total ion chromatogram (TIC) of VOCs; (**B**) Numbers of VOCs identified; (**C**) Relative content of different compound categories.

The analysis of VOCs from *S. commune* HJ-18 revealed a rich chemical diversity in its volatile metabolome. The relative abundance of each VOC was determined based on peak area percentages obtained from SPME-GC-MS analysis. In this study, a total of 53 VOCs were identified from the cultures of *S. commune* HJ-18 (Figure 2A). Based on structural and functional characteristics, the detected compounds primarily included alcohols, aldehydes, ketones, esters, nitrogen-containing heterocyclic compounds, as well as aromatic and hydrocarbon derivatives. In terms of the number of compounds identified, nitrogen-containing heterocyclic compounds and aromatic compounds along with their hydrocarbon derivatives were the most abundant, with 20 and 13 types, respectively. In contrast, alcohols, aldehydes, ketones, and esters were relatively fewer, with 6, 3, 4, and 7 types, respectively (Figure 2B).

However, from the perspective of relative abundance, alcohols constituted the most abundant class of VOCs, accounting for 39.78% of the total. Among these, 1-octen-3-ol, commonly known as “mushroom alcohol”, was particularly prominent. Its relative abundance alone reached 35.98%, representing 90.45% of the total alcohols, and it served as the primary contributor to the characteristic flavor profile of the strain. Aldehydes also represented a significant component, accounting for 24.63% of the total VOCs. These mainly included the pungent-smelling acrolein (2-Propenal) and (E)-2-octenal, which exhibits toxicity to fungi, with relative contents of 14.19% and 10.04%, respectively. Nitrogen-containing heterocyclic compounds accounted for 14.96% of the total VOCs. Although individual compounds within this class showed low abundance, the variety was extremely diverse. Esters were also relatively abundant, constituting 11.57% of the total, with short-chain esters being the most notable, such as 2-Propyn-1-ol propionate (6.57%). Ketones collectively accounted for 6.83%, with 2-decanone (4.97%) being a representative compound. Aromatic compounds and their hydrocarbon derivatives had the lowest relative abundance, accounting for only 2.23% (Figure 2C). The top 20 compounds by relative content accounted for 95.49% of the total VOCs (Table 1). In summary, alcohols and aldehydes together accounted for over 64% of the total VOCs, highlighting their prominence in the volatile profile. Given that 1-octen-3-ol, an alcohol, was confirmed to exhibit strong antifungal activity, these two classes may represent important contributors to the overall antifungal effect, although further bioassays of individual compounds are needed to fully elucidate their respective roles.

### 3.3. Calculation of the EC_50_ Concentration for 1-Octen-3-ol

**Figure 3:**
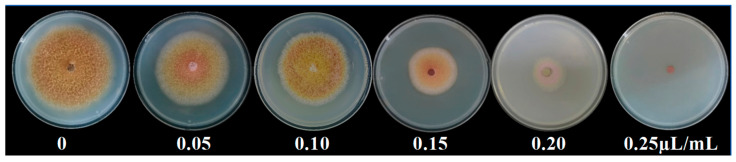
Effects of different concentrations of 1-octen-3-ol on the growth of *Fusarium tricinctum* colony after 9 d.

Given that 1-octen-3-ol was the most abundant compound identified in the VOCs and has been reported to possess significant antimicrobial potential, a specialized assessment was conducted to evaluate its inhibitory effect on the pathogenic fungus *F. tricinctum*. The concentration gradient of 1-octen-3-ol for antifungal testing was set at 0.05, 0.10, 0.15, 0.20, and 0.25 µL/mL. The results indicated that the inhibitory effect of 1-octen-3-ol on *F. tricinctum* was concentration-dependent. At a concentration of 0.25 µL/mL, the growth of *F. tricinctum* was completely inhibited (Figure 3). Linear regression analysis was performed using 1-octen-3-ol concentration as the independent variable (x) and mycelium growth inhibition rate as the dependent variable (y). The resulting regression equation was: y = 419.44x − 13.054 (95% CI for slope: 278.61 to 560.27; 95% CI for intercept: −27.74 to 1.63; R^2^ = 0.8934). Although dose–response relationships in biological systems often follow a sigmoidal pattern, linear regression was applied in this study because the inhibition rates within the concentration range covering the EC_50_ exhibited an approximately linear increase. The high coefficient of determination supports the adequacy of the linear model for estimating EC_50_ in this context. Therefore, the EC_50_ concentration of 1-octen-3-ol was calculated to be 0.15 µL/mL. This EC_50_ concentration was used for all subsequent experiments.

### 3.4. Inhibitory Effect of 1-Octen-3-ol on F. tricinctum

**Figure 4:**
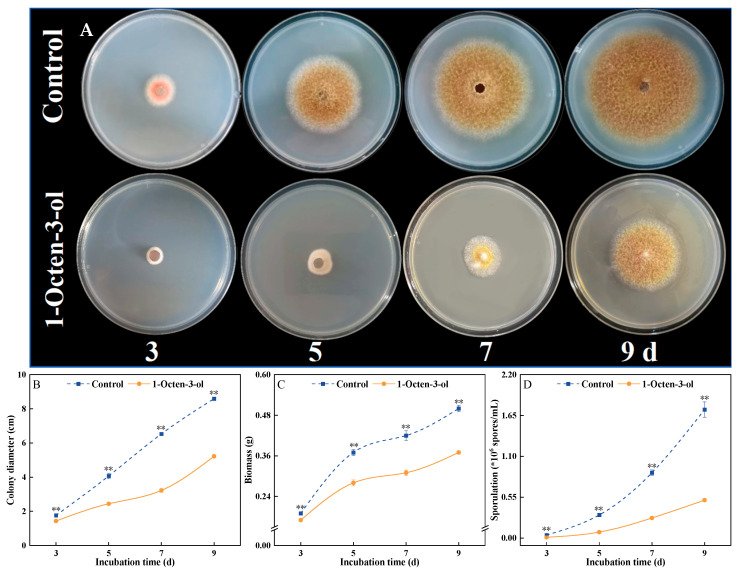
Effect of 1-octen-3-ol on colony growth (**A**), diameter (**B**), biomass (**C**), and sporulation (**D**) of *Fusarium tricinctum*. Values are presented as mean ± SE (*n* = 3). ** indicate significant differences at *p* < 0.01.

Under treatment with 1-octen-3-ol at the EC_50_ concentration, the growth of *F. tricinctum* was significantly inhibited, and this inhibitory effect intensified over time. In the early stages of incubation, the difference between the treated and control groups was minor, but it gradually increased during the mid to late incubation period. Colonies in the control group appeared yellowish-brown with dense mycelium, whereas colonies in the treated group exhibited lighter coloration and sparse mycelium (Figure 4A). Further analysis of key growth indicators confirmed this inhibitory effect. After 7 d of treatment, the colony diameter, mycelial biomass, and spore germination rate in the treated group were all significantly lower than those in the control group, with inhibition rates reaching 50.68%, 26.19%, and 69.31%, respectively (*p* < 0.05) (Figure 4B–D). These results indicate that 1-octen-3-ol effectively inhibits both mycelial growth and spore germination of *F. tricinctum*, thereby hindering its normal growth and development.

### 3.5. Effect of 1-Octen-3-ol on AKP and β-1, 3-Glucanase Activities

**Figure 5:**
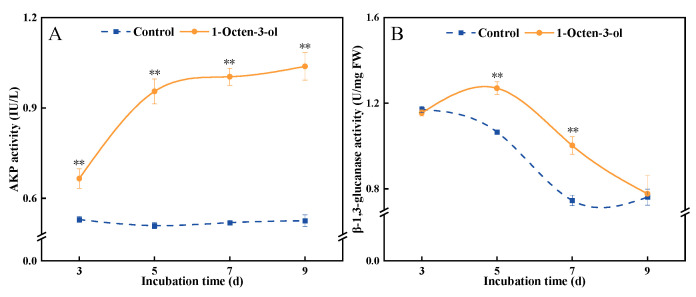
Effect of 1-octen-3-ol on AKP activity (**A**) and β-1, 3 glucanase activity (**B**) of *Fusarium tricinctum*. Values are presented as mean ± SE (*n* = 3). ** indicate significant differences at *p* < 0.01.

The activity of fungal AKP is associated with hyphal tip growth and cell wall synthesis and remodeling. During the incubation period, AKP activity in the control group remained relatively stable, whereas it gradually increased in the 1-octen-3-ol-treated group. Compared to the control, AKP activity in the treated group was higher throughout the entire incubation period. After 9 d of treatment, it was significantly higher than the control by 97.7% (Figure 5A).

β-1, 3-Glucan is a key structural component of the cell wall in the vast majority of fungi. High activity of β-1,3-glucanase can directly hydrolyze β-1,3-glucan chains in the fungal cell wall, compromising its structural integrity. The results showed that enzyme activity in the control group gradually decreased over the incubation time, while the 1-octen-3-ol-treated group exhibited an initial increase followed by a decrease. Compared to the control, β-1,3-glucanase activity in the treated group was higher throughout the entire incubation period. After 7 d of treatment, it was significantly higher than the control by 34.2% (Figure 5B). These findings suggest that 1-octen-3-ol treatment increased the activities of both AKP and β-1, 3-glucanase, and they may work synergistically in the degradation process of the *F. tricinctum* cell wall.

### 3.6. Effects of 1-Octen-3-ol on the Electrical Conductivity, Nucleic Acid, and Protein Leakage in F. tricinctum

**Figure 6:**
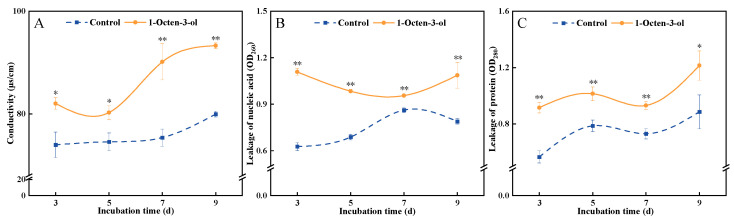
Effect of 1-octen-3-ol on conductivity (**A**), nucleic acid (**B**) and protein leakage (**C**) of *Fusarium tricinctum*. Values are presented as mean ± SE (*n* = 3). * and ** indicate significant differences at *p* < 0.05 and *p* < 0.01, respectively.

Electrical conductivity is a key indicator for assessing the integrity and permeability of fungal cell membranes. During the cultivation period, the electrical conductivity of the control group remained relatively stable, showing only a slight increase in the later stages. In contrast, the treatment group exhibited a trend of initial decrease followed by a rapid rise. Compared to the control, the electrical conductivity of the treatment group was consistently higher throughout the cultivation period. After 7 d of treatment, it was significantly higher than the control by 19.6% (Figure 6A). The level of protein leakage reflects the barrier capacity of the fungal cell membrane against macromolecular substances. During cultivation, the protein leakage in the control group initially increased slowly and then decreased slightly, whereas the treatment group consistently maintained a high level. Throughout the cultivation period, the protein leakage in the treatment group was higher than that in the control group. After 3 d of treatment, it was significantly higher than the control by 77.4% (Figure 6B). The level of nucleic acid leakage indicates the complete disintegration of the fungal cell membrane structure and the outflow of genetic material, typically signifying irreversible cell death. During cultivation, the changes in nucleic acid leakage were consistent between the treatment and control groups, both showing a slow upward trend overall. However, the nucleic acid leakage level in the treatment group was consistently higher than that in the control group. After 3 d of treatment, it was significantly higher than the control by 62.5% (Figure 6C). In summary, these findings indicate that treatment with 1-octen-3-ol caused severe damage to the structure and function of *F. tricinctum* cell membranes, increased permeability, and led to substantial leakage of cellular contents.

### 3.7. Effects of 1-Octen-3-ol on the O_2_^−^ Production Rate, and the Contents of H_2_O_2_ and ·OH in F. tricinctum

**Figure 7:**
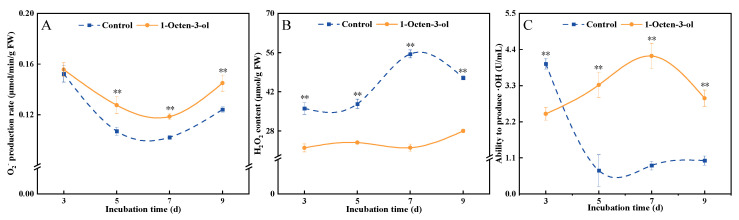
Effect of 1-octen-3-ol on O_2_^−^ production rate (**A**), H_2_O_2_ content (**B**) and ·OH content (**C**) of *Fusarium tricinctum*. Vertical bars represent standard error (±SE). Values are presented as mean ± SE (*n* = 3). ** indicate significant differences at *p* < 0.01.

The level of O_2_^−^ marks the initial stage of oxidative stress in fungi and is a key early signal of the ROS burst. During the cultivation period, the O_2_^−^ production rates in both the treatment and control groups showed a trend of initial decrease followed by an increase. Compared to the control, 1-octen-3-ol treatment induced an increase in the O_2_^−^ production rate, which remained higher than the control during the middle and later stages of cultivation. After 7 d of treatment, it was significantly higher than the control by 15.6% (Figure 7A). H_2_O_2_ represents the accumulated and relatively stable state of ROS. Its strong permeability and relative stability enable it to cause widespread oxidative damage. The results revealed that the H_2_O_2_ content in the control group first increased and then decreased, whereas the H_2_O_2_ content in the treatment group remained at a low level and was lower than the control throughout the entire cultivation period (Figure 7B). ·OH is the most reactive oxygen radical, and its content represents the ultimate degree of oxidative damage, capable of indiscriminately destroying nearly all biological macromolecules. During cultivation, the ·OH content in the treatment group initially decreased rapidly and then remained relatively stable. In contrast, the ·OH content in the treatment group showed a trend of first increasing and then decreasing, and it remained higher than the control group during the middle and later stages of cultivation. After 7 d of treatment, it was significantly higher than the control by 3.88-fold (Figure 7C). The above results indicate that 1-octen-3-ol treatment induced an increase in the O_2_^−^ production rate, consumed H_2_O_2_, and promoted the conversion and accumulation of ROS, leading to an increase in ·OH content.

### 3.8. Effects of 1-Octen-3-ol on the Antioxidant Levels in F. tricinctum

**Figure 8:**
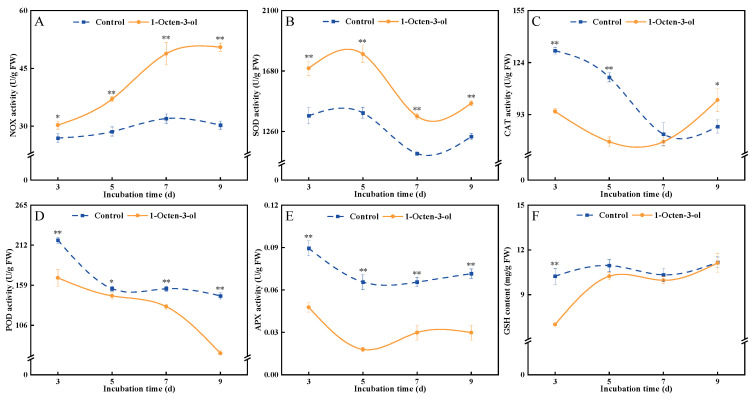
Effect of 1-octen-3-ol on NOX (**A**), SOD (**B**), CAT (**C**), POD (**D**), APX (**E**), and GSH (**F**) activities of *Fusarium tricinctum*. Values are presented as mean ± SE (*n* = 3). * and ** indicate significant differences at *p* < 0.05 and *p* < 0.01, respectively.

NOX is a key enzyme that catalyzes the production of O_2_^−^ and initiates the cellular oxidative burst. During the cultivation period, the NOX activity in the control group increased slightly in the later stages but remained at a low level overall. In contrast, the treatment group showed a trend of rapid increase, and its NOX activity was consistently higher than that of the control group throughout the cultivation period. After 7 d of treatment, it was significantly higher than the control by 52.6% (Figure 8A). SOD rapidly converts the more toxic O_2_^−^ into H_2_O_2_ and serves as the first critical line of defense in the fungal antioxidant system. The SOD activities in both the control and treatment groups exhibited a trend of initial increase, followed by a decrease, and then another increase. However, the activity in the treatment group was consistently higher than that in the control group, and after 7 d of treatment, it was significantly higher than the control by 23.4% (Figure 8B). CAT efficiently scavenges high concentrations of H_2_O_2_ by decomposing it into water and oxygen. During cultivation, the CAT activities in both the control and treatment groups showed a trend of initial decrease followed by a slight increase. Compared to the control, 1-octen-3-ol treatment reduced CAT activity in the early stages of cultivation. After 5 d of treatment, it was significantly lower than the control by 33.3% (Figure 8C). POD could utilize various substrates (such as phenols and amines) to reduce and scavenge H_2_O_2_. The POD activities in both the control and treatment groups gradually decreased during the cultivation period. The POD activity in the treatment group was consistently lower than that in the control group, and after 9 d of treatment, it was significantly lower than the control by 52.4% (Figure 8D). APX specifically uses AsA as an electron donor to scavenge H_2_O_2_ and is a key enzyme in maintaining cellular redox balance. The results showed that the trends in APX activity were similar between the treatment and control groups during cultivation, but the activity in the treatment group was significantly lower than that in the control. After 5 d of treatment, it was significantly lower than the control by 55.3% (Figure 8E). GSH could both directly neutralize free radicals and serve as a substrate for enzymes like APX to participate in H_2_O_2_ scavenging. The GSH content in the control group showed no significant change during cultivation, but 1-octen-3-ol treatment reduced the GSH content in the early stages. After 5 d of treatment, it was significantly lower than the control by 31.5% (Figure 8F). In summary, these results indicate that 1-octen-3-ol treatment induced an increase in NOX and SOD activities but inhibited the activities of CAT, POD, and APX, while also reducing the GSH content.

## 4. Discussion

VOCs produced by fungi have been extensively studied as antimicrobial agents [22]. This study confirms that the VOCs released by the *S. commune* HJ-18 strain could significantly inhibit the pepper gummosis pathogen *F. tricinctum*. To further elucidate the material basis of the antimicrobial activity of *S. commune* HJ-18, we identified the components of its VOCs, detecting a total of 53 compounds spanning categories such as alcohols, aldehydes, nitrogen-containing heterocyclic compounds, esters, and ketones. Among these, alcohols dominated in relative abundance (39.78%), with 1-octen-3-ol emerging as the key characteristic component due to its absolute predominance at 35.98% (Figure 2, Table 1). This finding aligns with the typical volatile profiles of other mushroom fungi, where 1-octen-3-ol is often recognized as the primary contributor to the “mushroom odor” [23]. Furthermore, aldehydes present in relatively high concentrations, such as acrolein and trans-2-octenal, have also been reported to possess some fungal toxicity and may play a synergistic role in the overall antimicrobial effect. In addition, the remaining categories, including esters (11.57%), nitrogen-containing heterocyclic compounds (14.96%), ketones (6.83%), and aromatic compounds (2.23%), enriched the chemical diversity and may contribute to auxiliary or synergistic biological roles. Based on its high relative abundance and widely reported antimicrobial activity [24], 1-Octen-3-ol was selected as the key compound for further mechanistic investigation. Previous studies have reported the antifungal activity of 1-Octen-3-ol against various pathogens, including *Escherichia coli*, *Monilinia fructicola*, and *F. oxysporum* [25,26]. These studies primarily attributed its antifungal effect to disruption of the cell membrane lipid bilayer. In contrast, the present study provides a more comprehensive mechanistic framework by demonstrating that 1-octen-3-ol not only compromises membrane integrity but also disrupts cell wall structure and induces a distinctive oxidative stress response.

The cell wall and cell membrane are core barriers maintaining fungal cell structural stability and physiological functions. The cell wall provides rigid protection, while the cell membrane regulates material exchange and signal transduction, together constituting the first line of defense against external stress [27]. AKP is an enzyme located in the periplasmic space between the cell membrane and cell wall, and its abnormal elevation is often considered a marker of increased permeability or damage to the cell wall [28]. β-1, 3-glucanase could hydrolyze β-1, 3-glucan, a key structural component of the fungal cell wall [29]. 1-octen-3-ol treatment significantly increased the activities of AKP and β-1, 3-glucanase in *F. tricinctum* (Figure 5), suggesting it may interfere with the normal synthesis and repair of the cell wall, thereby making its structure fragile and laying the groundwork for subsequent antimicrobial effects. The cell membrane is a classical target for lipophilic small-molecule compounds like 1-octen-3-ol. Changes in electrical conductivity and the leakage of intracellular macromolecules such as nucleic acids and proteins are key indicators for assessing the integrity and functional state of the fungal cell membrane. In this study, we similarly observed that after 1-octen-3-ol treatment, the electrical conductivity of the mycelium, as well as the leakage of intracellular nucleic acids and proteins, significantly increased (Figure 6). These changes directly reflect compromised selective permeability and loss of the ability to maintain ionic balance and prevent outflow of intracellular contents. Notably, the concurrent increase in ·OH levels (discussed below) suggests that oxidative stress induced by 1-Octen-3-ol directly contributes to membrane damage, consistent with the known ability of ·OH to initiate lipid peroxidation and disrupt membrane integrity [30]. Similarly, VOCs produced by *Pseudomonas fluorescens* also inhibit *B. cinerea* by disrupting cell membrane integrity, increasing membrane permeability, and causing leakage of cellular contents [31]. Therefore, we propose that 1-octen-3-ol treatment disrupts the cell wall integrity of *F. tricinctum* and leads to increased cell membrane permeability. These results are consistent with previous findings that microbial VOCs could damage the cell walls and membranes of *A. alternata*, *A. flavus*, and *F. oxysporum* [32,33,34].

ROS maintain a dynamic balance within fungal cells and participate in normal signaling and metabolic processes. However, when ROS in fungi accumulate excessively due to external stress, it can trigger severe oxidative damage, destroying key cellular components such as proteins, lipids, and nucleic acids, ultimately leading to cell death [35]. Therefore, inducing excessive ROS accumulation has become a key mechanism through which various antimicrobial substances exert their inhibitory effects [36]. O_2_^−^ is one of the earliest forms of ROS generated within cells and is a critical starting point for initiating subsequent cascades of oxidative damage. ·OH is known to be the most reactive and destructive type of ROS, capable of indiscriminately attacking nearly all biological macromolecules, such as proteins, lipids, DNA, and polysaccharides [30]. In this study, we found that 1-octen-3-ol treatment significantly increased the O_2_^−^ production rate and ·OH content in *F. tricinctum*, while concurrently depleting H_2_O_2_ (Figure 7). This alteration in ROS profile may stem from multiple factors. The significant enhancement of NOX activity directly drove the generation of O_2_^−^. The compensatory increase in SOD activity then converted O_2_^−^ into H_2_O_2_. However, the key antioxidant enzyme systems responsible for scavenging H_2_O_2_, including CAT, POD, and APX-as well as the important antioxidant GSH, were all significantly inhibited or reduced by 1-octen-3-ol treatment (Figure 8). This scenario of “production without clearance” would typically lead to H_2_O_2_ accumulation. Interestingly, however, we did not observe H_2_O_2_ accumulation but detected a significant increase in ·OH content. Previous studies have shown that H_2_O_2_ can be converted into ·OH via Fenton-type reactions catalyzed by metal ions, leading to severe oxidative damage to cells [37]. In the present study, treatment with 1-octen-3-ol resulted in decreased H_2_O_2_ levels and a concurrent increase in ·OH levels. Although direct evidence of metal ion involvement is lacking, it is possible that the observed changes may be attributed to a Fenton-like reaction. We therefore hypothesize that 1-octen-3-ol may promote the conversion of H_2_O_2_ to ·OH, thereby contributing to oxidative damage, and ultimately pathogen death.

Collectively, these findings extend previous knowledge on the antifungal activity of 1-octen-3-ol by providing a more detailed mechanistic framework. While earlier studies primarily focused on membrane disruption, our results reveal that 1-octen-3-ol also compromises cell wall integrity and induces a distinctive oxidative stress response characterized by H_2_O_2_ depletion and ·OH accumulation. This comprehensive mode of action—involving multi-target effects on cell wall, membrane, and redox balance—has not been previously reported for 1-octen-3-ol and offers a deeper understanding of its antifungal potential. It is important to acknowledge that all experiments in this study were conducted under in vitro conditions, which may not fully reflect the complexity of agricultural systems. The EC_50_ value of 1-octen-3-ol was determined under such controlled conditions; however, in natural VOC mixtures, synergistic interactions among multiple compounds may enhance overall antifungal activity, potentially reducing the concentration required for effective inhibition. Despite these limitations, our findings position *S. commune* as a promising VOC-producing biocontrol agent. As an endophytic fungus, it has the potential to colonize plant tissues and continuously release antifungal VOCs in situ. Additionally, 1-octen-3-ol—a food-grade flavor compound with high volatility—holds promise for development as a natural fumigant for managing pepper gummosis, particularly in postharvest and protected cultivation settings. Future studies should focus on evaluating the efficacy of *S. commune* and its VOCs under in planta and field conditions, along with optimizing application methods to ensure consistent disease control in practice.

## 5. Conclusions

**Figure 9:**
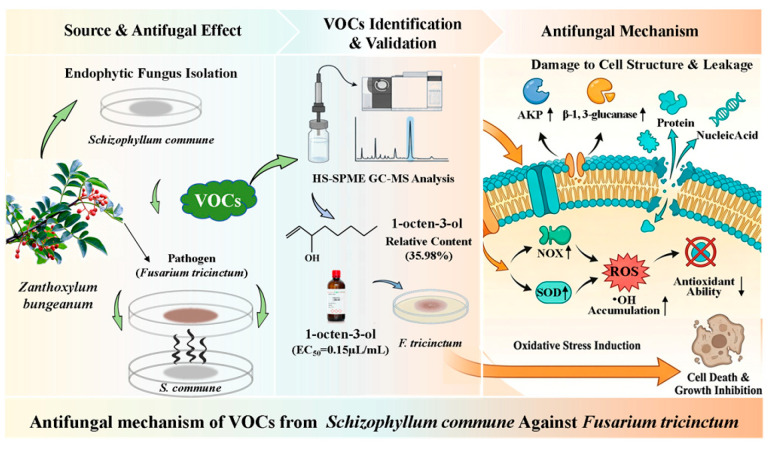
A possible mechanism diagram of the antifungal effect of 1-octen-3-ol.

This study demonstrates that VOCs released by the *Zanthoxylum*-endophytic fungus *Schizophyllum commune* effectively inhibit the growth of *Fusarium tricinctum*, the causal agent of pepper gummosis. Among these VOCs, 1-octen-3-ol is likely a major contributor to the antifungal activity, and its inhibitory mechanism against *F. tricinctum* is associated with disrupting cell wall integrity, increasing membrane permeability, and inducing oxidative stress. Based on these findings, a schematic diagram was proposed to illustrate the proposed antifungal mechanism (Figure 9). This research provides a theoretical foundation and a technical approach for the green control of pepper gummosis using microbial VOCs.

## Figures and Tables

**Table 1:** Identification and relative content of VOCs released by *S. commune* HJ-18 (Top 20).

Retention Time (min)	Volatile Organic Compounds	Compound Class	Relative Content (%)	Chemical Abstracts Service Number	Molecular Formula
8.62	1-octen-3-ol	Alcohol	35.98	3391-86-4	C_8_H_16_O
11.09	2-Propenal	Aldehyde	14.19	107-02-8	C_3_H_4_O
10.64	2-Octenal, (E)	Aldehyde	10.04	2548-87-0	C_8_H_14_O
11.07	1H-Pyrrole, 2, 5-dihydro	Nitrogen-containing heterocyclic	9.87	109-96-6	C_4_H_7_N
8.40	2-Propyn-1-ol, propionate	Ester	6.57	1932-92-9	C_6_H_8_O_2_
11.07	1,1-Diethylpropargylamine	Nitrogen-containing heterocyclic	2.91	3234-64-8	C_7_H_13_N
11.10	Bicyclo[4.1.0]heptane, 7-methylene	Aromatic and hydrocarbon derivatives	1.92	54211-14-2	C_8_H_12_
10.66	Furan, 2-ethyl-5-methyl	Aromatic and hydrocarbon derivatives	1.57	1703-52-2	C_7_H_10_O
8.46	1-Hexen-3-ol	Alcohol	1.50	4798-44-1	C_6_H_12_O
10.99	2-Cyclopenten-1-one, 3-ethyl	Ketone	1.41	5682-69-9	C_7_H_10_O
8.51	3,6-Heptanedione	Ketone	1.40	1703-51-1	C_7_H_12_O_2_
10.95	3-Hexen-1-ol, propanoate, (Z)	Ester	1.34	33467-74-2	C_9_H_16_O_2_
11.00	cis-3-Hexenyl-alpha-methylbutyrate	Ester	1.03	53398-85-9	C_11_H_20_O_2_
4.79	1,2,4-Benzenetricarboxylic acid, 1,2-dimethyl ester	Ester	1.02	54699-35-3	C_11_H_10_O_6_
8.93	4-Methyl-5-decanol	Alcohol	1.01	213547-15-0	C_11_H_24_O
8.61	1,4-Dioxane-2,6-dione	Aromatic and hydrocarbon derivatives	0.92	4480-83-5	C_4_H_4_O_4_
8.55	1H-Tetrazole, 1-methyl	Nitrogen-containing heterocyclic	0.81	16681-77-9	C_2_H_4_N_4_
8.70	3-Octanone	Ketone	0.77	106-68-3	C_8_H_16_O
22.92	2,4-Di-tert-butylphenol	Aromatic and hydrocarbon derivatives	0.63	96-76-4	C_14_H_22_O
8.39	2-Pentanone, 1-phenyl	Ketone	0.60	6683-92-7	C_11_H_14_O

## Data Availability

The raw data supporting the conclusions of this article will be made available by the authors on request.
